# A Scoping Review of the Positive and Negative Bacteria Associated With the Gut Microbiomes of Systemic Lupus Erythematosus Patients

**DOI:** 10.7759/cureus.57512

**Published:** 2024-04-03

**Authors:** Marissa N McPhail, Michael Wu, Kelsey Tague, Hassaan Wajeeh, Michelle Demory Beckler, Marc M Kesselman

**Affiliations:** 1 Osteopathic Medicine, Nova Southeastern University Dr. Kiran C. Patel College of Osteopathic Medicine, Fort Lauderdale, USA; 2 Microbiology and Immunology, Nova Southeastern University Dr. Kiran C. Patel College of Allopathic Medicine, Fort Lauderdale, USA; 3 Rheumatology, Nova Southeastern University Dr. Kiran C. Patel College of Osteopathic Medicine, Davie, USA

**Keywords:** autoimmune disease, autoimmune gut microbiome, gastrointestinal microbiome, gut microbiomes, gut microbiota dysbiosis, sle pathogenesis, systemic lupus erythematosis

## Abstract

Systemic lupus erythematosus (SLE) is a chronic autoimmune disease that affects multiple systems of the body. Recent research on the gut microbiota dysbiosis associated with SLE patients has gained traction and warranted further exploration. It has not been determined whether the change in the gut microbiota is a cause of SLE or a symptom of SLE. However, based on the physiological and pathophysiological role of the bacteria in the gut microbiome, as levels of the bacteria rise or fall, symptomatology in SLE patients could be affected. This review analyzes the recent studies that examined the changes in the gut microbiota of SLE patients and highlights the correlations between gut dysbiosis and the clinical manifestations of SLE. A systematic search strategy was developed by combining the terms "SLE," “systemic lupus erythematosus," and "gut microbiome." Biomedical Reference Collection, CINAHL, Medline ProQuest, and PubMed Central databases were searched by combining the appropriate keywords with "AND." Only full-text, English-language articles were searched. The articles were restricted from 2013 to 2023. Only peer-reviewed controlled studies with both human and animal trials were included in this scoping review. Review articles, non-English articles, editorials, case studies, and duplicate articles from the four databases were excluded. Various species of bacteria were found to be positively or negatively associated with SLE gut microbiomes. Among the bacterial species increased were *Clostridium, Lactobacilli, Streptococcus, Enterobacter, *and* Klebsiella*. The bacterial species that decreased were *Bifidobacteria, Prevotella, *and the *Firmicutes/Bacteroidetes *ratio. Literature shows that *Clostridium* is one of several bacteria found in abundance, from pre-disease to the diseased state of SLE. *Lachnospiraceae* and *Ruminococcaceae *are both part of the family of butyrate-producing anaerobes that are known for their role in strengthening the skin barrier function and, therefore, may explain the cutaneous manifestations of SLE patients. Studies have also shown that the *Firmicutes/Bacteroidetes* ratio is significantly depressed, which may lead to appetite changes and weight loss seen in SLE patients. Based on the established role of these bacteria within the gut microbiome, the disruption in the gut ecosystem could explain the symptomatology common in SLE patients. By addressing these changes, our scoping review encourages further research to establish a true causal relationship between the bacterial changes in SLE patients as well as furthering the scope of microbiota changes in other systems and autoimmune diseases.

## Introduction and background

Systemic lupus erythematosus (SLE) is a chronic autoimmune disease that affects multiple body systems, including the dermatological, renal, neuropsychiatric, and cardiovascular systems. It can be identified through several different presentations and diagnoses confirmed by the presence of four of the eleven criteria detailed in the 1997 American College of Rheumatology (ACR) criteria [1]. These criteria include malar rash, discoid rash, photosensitivity, alopecia, oral/nasal ulcers, arthritis, serositis, renal disease, hematologic disease, neurologic disease, immunologic criteria, and/or antinuclear antibody (ANA) positivity in the absence of known drugs to cause drug-induced lupus [1]. The Centers for Disease Control and Prevention (CDC) report a conservative estimate that suggests a prevalence of 161,000 with definite SLE and 322,000 with definite or probable SLE [2]. While the exact pathophysiology of SLE remains ambiguous, multifactorial interactions between genetic and environmental factors have been proposed among numerous causes. Women more than men, hormonal risk (estrogen and prolactin), and environmental triggers have all demonstrated a strong positive correlation to an increased risk of SLE.

Recently, dysbiosis of the gut microbiota has been discovered to be associated with SLE; however, it has not yet been determined whether observed changes in the gut microbiota are a cause of SLE, a symptom of SLE, or a result of SLE. However, based on the role of bacteria in the normal gut microbiome, as levels of the bacteria rise or fall, observed dysbiosis in SLE could explain some of the symptomatology exhibited by SLE patients. With the consideration of regulating the gut microbiome, there is a potential to give some autonomy to SLE patients in relieving some of their signs and symptoms with dietary and nutrient changes. The goal of this review is to summarize the changes that have been found in the gut microbiota and highlight the correlations between dysbiosis of the gut microbiota and the clinical manifestations of SLE.

Relationship between SLE, the gut microbiome, and the immune system

One essential component for the diagnosis of SLE that serves as the entry criterion for the European Alliance of Associations for Rheumatology (EULAR)/ACR classification criteria is an ANA titer of ≥1:80 on HEP-2 cells or an equivalent positive test [3]. Other auto-reactive antibodies characteristic of SLE include anti-double-stranded DNA (anti-dsDNA) antibodies, anti-Smith (SM) antibodies, and anti-Sjogren’s antibodies [4]. The dysregulation of the adaptive immune network in SLE can induce the production of high levels of these antibodies due to abnormal T- and B-cell activation and impaired apoptotic pathways [5]. While the exact pathogenesis underlying the dysregulation of the adaptive immune system in SLE is unknown, it has been postulated that inflammation is the foundation of the disease. Current literature suggests the possibility that one primary factor contributing to the onset and progression of inflammatory autoimmune conditions, such as SLE, type I diabetes, and rheumatoid arthritis, is microbiome dysbiosis [5]. This dysbiosis appears to increase bowel permeability, which causes bacterial products to leak out from the gut, resulting in inflammation and, therefore, an overactive immune system. In a patient with elevated ANAs and a weak ability to prevent reactivity to self-antigens, a leaky gut may be the final factor leading to the manifestation of the clinical hallmarks of SLE [5].

The gut microbiota occupies the mucus layer lining the digestive tract and is important in inhibiting colonization and invasion of gastrointestinal pathogens. About 70-80% of human immune cells are in the gut, making the gut microbiome and immune system deeply interconnected on many levels [6]. For one, the gut microbiome is essential in the development of gut-associated lymphatic tissue (GALT). GALT protects against mucosal pathogens and allows tolerance to self-antigens and commensal bacteria. The gut microbiome is also key in the development of regulatory T cells and helper T cells [5]. Regulatory T cells help regulate the gut's beneficial and pathogenic bacteria. Depletion of regulatory T cells can lead to microbial dysbiosis and loss of tolerance to commensal bacteria [4].

Helper T cells, on the other hand, can assist in immune responses to pathogens by secreting cytokines such as interleukin-17 (IL-17) and interleukin-22 (IL-22), cytokines critical for the recruitment of leukocytes [7]. Additionally, helper T cells are important in B-cell activation into immunoglobulin A (IgA)-secreting plasma cells with high antigen affinity. IgA coats the mucosal surface of our guts and can tightly bind pathogenic bacteria and weakly bind commensal bacteria. This allows for the elimination of pathobionts and the maintenance of beneficial microbiota. Together, the gut microbiome and immune system concomitantly produce both innate and adaptive immune responses that protect, limit, and eliminate pathogenic species from the gut. In addition, this coordinated action appears to limit leaky gut occurrences, aiding in the prevention of inflammatory reactions underlying autoimmune diseases such as SLE [7]. However, it is still unknown whether the gut dysbiosis seen in SLE is a consequence of the disease or a cause. This scoping review begins to assess the current literature on microbiota changes in the gut in SLE patients.

## Review

Methodology 

Study Selection Process

**Figure 1:**
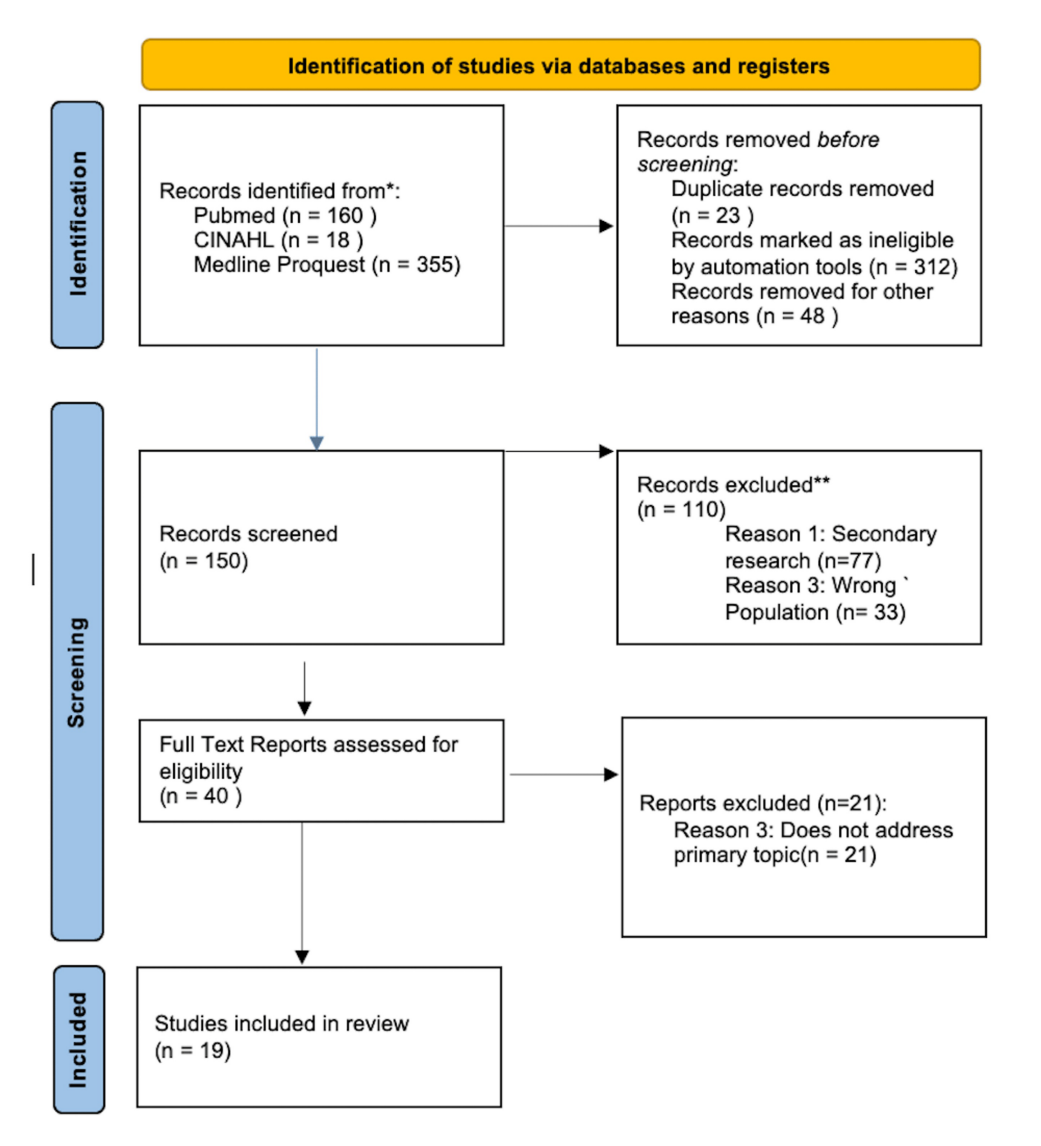
PRISMA diagram describing screening method for article selection.

Data collection took place in May 2023 in the following databases: PubMed, Ovid MEDLINE, and CINAHL. The research question was created using the Population, Concepts, and Context (PCC) strategy. The population is animals or adults 18 years and older with SLE, with concepts and context being the changes in the gut microbiome in people and animals with SLE and outpatient settings (U.S. and international). Considering these parameters, our research question used for the data collection was, “How did the gut microbiome change in SLE patients, and what is the correlation between the gut microbiome changes and the symptomatology of SLE?”. The database search was conducted using the Boolean operators “AND” and “OR” between the keywords selected for the literature search as follows: (SLE) OR (systemic lupus erythematosus) AND (gut microbiome). The articles were filtered to be within 2010-2023 in the English language, and relevance was evaluated using a hierarchical approach that evaluated the title, abstract, and then the full manuscript. The studies included were considered eligible if they were investigated through randomized or nonrandomized control trials. The inclusion criteria incorporated the English language and free full-text articles, articles including participants aged 18 years and older, bacterial gut microbiomes, and the inclusion of keywords within the title and abstract. Human and animal studies from all continents were both included in the scoping review. Exclusion criteria included study designs, which were secondary analysis-based studies, literature reviews, the inaccessibility of the full-text version, and duplicate studies. Review articles, non-English articles, editorials, case studies, and duplicate articles from the three databases were excluded. Articles referencing pediatric populations, viral genomes, oral genomes, and other autoimmune diseases were excluded. The results of the search and the study inclusion process were presented in a Preferred Reporting Items for Systematic Reviews and Meta-analyses extension for scoping review (PRISMA-ScR) flow diagram (Figure 1) and reported in full in the final review.

Study Selection Results

The initial search revealed 533 relevant citations; initially, 26 duplicate citations were removed, 312 articles were removed for non-peer review, and 48 articles were removed for non-availability of full text or non-English. The remaining 150 articles were assessed for eligibility. The authors proceeded to determine the eligible articles for further analysis of the proper inclusion criteria (n=150 articles). Of these articles, n=110 were excluded for not being primary research (n=77) and the wrong autoimmune patient population being studied (n=33). The wrong population included pediatric populations, patients diagnosed with other autoimmune conditions, or lupus nephritis. We identified 40 articles that underwent full-text screening for eligibility; n=21 articles were removed for not addressing the primary topic. After the removal of articles not within the inclusion criteria, n=19 studies were presented in this review.

Results

All recent literature and studies included have been conducted recently, mostly in the last decade of research; therefore, this study serves to provide a scoping review of the topic of gut microbiota changes in SLE patients. With the small scope of available research, we have included human and animal studies from all countries to review the most pertinent information regarding this topic. There were twelve human studies, five animal studies, and two human and animal cohort studies included in this review. The included studies are delineated in Table 1 and organized into categories based on the type of study conducted, positive bacteria identified, negative bacteria identified, and pertinent findings of each study.

**Table 1:** Results table displaying summary of review findings of positive and negatively associated bacteria associated with systemic lupus erythematosus patients. n/a: not applicable, SLE: systemic lupus erythematosus.

Author	Title of study	Type of study	Positive bacteria	Negative bacteria	Pertinent findings
Chen et al. [7]	An Auto-Immunogenic and Proinflammatory Profile Defined by the Gut Microbiota of Patients with Untreated Systemic Lupus Erythematosus	Cohort human and animal study	Clostridium, Atopobium rimae, Shuttleworthia satelles, Actinomyces massiliensis, Clostridium leptum, and Bacteroides fragilis	n/a	This study found approaches to alternating the microbiome may serve as adjuvant treatments for SLE.
Xiang et al. [8]	Causal Effects of Gut Microbiome on Systemic Lupus Erythematosus: A Two-Sample Mendelian Randomization Study	A two-sample Mendelian randomization study in human patients.	Bacilli, Lactobacillales	Bacillales, Coprobacter, Lachnospira, Ruminoclostridium	The causal effects of gut microbiome components on SLE risk are indicated by the results in this study.
Tomofuji et al. [9]	Metagenome-wide association study revealed disease-specific landscape of the gut microbiome of systemic lupus erythematosus in Japanese.	Genome-wide association study in human patients	Streptococcus intermedius, Streptococcus anginosus	n/a	This study found that belonging to normal oral and gastrointestinal cavity flora in the *Streptococcus anginosus* group, oral-gut interaction mediated by microbes suggests a possible oral-gut axis and SLE association.
He et al. [10]	Microbiome and Metabolome Analyses Reveal the Disruption of Lipid Metabolism in Systemic Lupus Erythematosus	Gut microbiome sequencing and metabolomics studies in human patients	Proteobacteria, Enterobacteriaceae, Escherichia, Shigella	Bacteroidetes, Clostridia, Ruminococcaceae, Faecalibacterium	This study found microbial metabolism disruption in SLE patients may contribute to the pathogenesis of SLE and its multi-system organ involvement.
Choi et al. [11]	Gut microbiota dysbiosis and altered tryptophan catabolism contribute to autoimmunity in lupus-susceptible mice.	Metabolomic animal studies	Prevotellaceae, Paraprevotella, Lactobacillus, Bifidobacterium, Allobaculum, Staphylococcus xylosus	n/a	This study showed the aberrant metabolism of tryptophan due to gut dysbiosis may lead to the autoimmune activation of SLE patients.
Zhang et al. [12]	Dynamics of gut microbiota in autoimmune lupus	Metagenomic animal studies	Lachnospiraceae, Clostridiales, Streptococcaceae	Lactobacillaceae	This study provides evidence for the use of dietary supplements consisting of probiotic *lactobacilli* and retinoic acid to relieve inflammatory flares.
Toumi et al. [13]	Gut microbiota in systemic lupus erythematosus patients and lupus mouse model: a cross-species comparative analysis for biomarker discovery	Cross-sectional human and animal studies	Tannerellaceae Alistipes, Flintibacter, Parabacteroides Desulfovibrio piger, Bacteroides, Ruminococcus gnavus	Tenericutes, Firmicutes/Bacteroidetes ratio, Bacillicaceae, Clostridiales, Ruminococcaceae, Eubacteriaceae, Lactobacillaceae, Romboutsia, Lactobacillus, Fusicatenibacter, Turicibacter, Faecalibacterium prausnitzii, Fusicatenibacter saccharivorans, and Eubacterium cellulosomes	This study concluded *Ruminococcaceae, Bifidobacterium, and Ruminococcus gnavus* may play an essential role in SLE severity. A better understanding of this mechanism may provide a target for future studies into the treatment of SLE.
Luo et al. [14]	Gut Microbiota in Human Systemic Lupus Erythematosus and a Mouse Model of Lupus	Experimental animal study	Clostridium, Dehalobacterium, Lactobacillus, Oscillospira, Dorea, Bilophila, Ruminococcaceae	Akkermansia muciniphila and Anaerostipes (family Lachnospiraceae)	This study found that *lactobacillaceae and lactobacillus* significantly increased from pre-disease onset to post-disease onset as well as had a positive correlation to renal function and systemic autoimmunity.
He et al. [15]	Alterations of the gut microbiome in Chinese patients with systemic lupus erythematosus	Cross-sectional human study	Rhodococcus, Eggerthella, Klebsiella, Prevotella, Eubacterium, Flavonifractor, Incertae sedis	Dialister and Pseudobutyrivibrio	This study found alterations of the gut microbiome were consistent at the phylum, family, and genus level. Therefore, this study showed altered genera in SLE patients may be used to discriminate between SLE and healthy controls.
Li et al. [16]	Disordered intestinal microbes are associated with the activity of systemic lupus erythematosus	Metabolomic study using PICRUSt analysis in human patients	Streptococcus, Campylobacter, Veillonella, anginosus and dispar species	Bifidobacterium	This study showed that there was a particular distinction in active SLE patients with significant dysbiosis, reduced bacterial diversity, and biased community constitutions.
Liu et al. [17]	Distinct Microbiomes of Gut and Saliva in Patients with Systemic Lupus Erythematosus and Clinical Associations	Cross-sectional study of human patients	Lactobacillus	Ruminococcaceae, Bifidobacterium	Significantly reduced levels of bacterial richness and diversity was found in all stages of SLE patients regardless of the level of disease activity.
Van der Meulen et al. [18]	Shared gut, but distinct oral microbiota composition in primary Sjögren's syndrome and systemic lupus erythematosus	Cross-sectional study of human patients	Bacteroides	*Firmicutes/Bacteroidetes *ratio	*Bacteroides* species are important for glycan degrading and short-chain fatty acid production which is considered beneficial to the host therefore high abundance of *Bacteroides* is not directly associated with microbiome dysbiosis.
Wei et al. [19]	Changes of intestinal flora in patients with systemic lupus erythematosus in northeast China	Cross-sectional study of human patients	Firmicutes, Bacteroidetes and Proteobacteria, Enterobacteriaceae, Streptococcus	Ruminococcaceae, Prevotellaceae, Clostridiales, Prevotella, Roseburia, Ruminococcaceae, Paraprevotella, Ezakiella	When comparing the results of their study to other studies conducted, they found that the unique geographical location and dietary habits may play a role in differences in the gut microbiota of SLE patients in different countries.
Xu et al. [20]	Causal Relationship Between Gut Microbiota and Autoimmune Diseases: A Two-Sample Mendelian Randomization Study	A two-sample Mendelian randomization study in human patients.	Ruminococcus	Bifidobacterium	This study determined that an increased abundance of the bacterial genus *Bifidobacterium* was associated with a lower risk of SLE which contrasts with the higher risk associated with multiple sclerosis, type 1 diabetes and Celiacs disease. *Ruminococcus* level was associated with a higher risk of SLE.
Ma et al. [21]	Lupus gut microbiota transplants cause autoimmunity and inflammation	Cross-sectional study in human patients	Turicibacter, Clostridium papyrosovens, Lactobacillus reuteri, Lactobacillus intestinalis, Lachnospiraceae bacterium A2, Lachnospiraceae, Bacterium M18-1	n/a	*Clostridia, Clostridiales, Lachnospiraceae, Lachnospiraceae, Subdoligranulum* were significantly enriched in the human control group indicating that these biomarkers are important to maintain a healthy state of being.
Ma et al. [22]	Gut microbiota promotes the inflammatory response in the pathogenesis of systemic lupus erythematosus.	Experimental animal study	Firmicutes phylum (Erysipelotrichia, Erysipelotrichales, Erysipelotrichaceae, and Turicibacter) and Actinobacteria phylum	n/a	This study determined that while genetic defects and environmental factors can trigger severe SLE, intestinal microbiota alone can induce some of the symptoms of SLE due to peripheral and mucosal immune system influences.
Zhang et al. [23]	The level of peripheral regulatory T cells is linked to changes in gut commensal microflora in patients with systemic lupus erythematosus.	Correlational analysis in human patients.	Proteobacteria, Bacteroidetes and Actinobacteria Bacteroidaceae, Veillonellaceae, Klebsiella, Streptococcaceae, Erysipelotrichaceae, Ruminococcus genus	Firmicutes, Ruminococcaceae	This study found that the changes in taxonomic diversity and abundance of specific flora may play a role in the number of T regulatory cells in peripheral blood which participates in the pathogenesis of SLE.
Yi et al. [24]	Fecal microbiota from MRL/lpr mice exacerbates pristane-induced lupus	Metabolomic animal study followed by correlation analysis	Bacteroides, Prevotella	*Firmicutes/Bacteroidetes *ratio	This study found that several strains of *Prevotella *may serve as a biomarker for lupus aggravated states.
Gerges et al. [25]	Altered Profile of Fecal Microbiota in Newly Diagnosed Systemic Lupus Erythematosus Egyptian Patients.	Cross-sectional human study	Bacteroidetes	*Phylum Firmicutes, Firmicutes/Bacteroidetes *ratio*, Lactobacillus*	This study found a negative correlation between SLE disease activity and *lactobacillus* abundance, however, it was not significant.

**Figure 2:**
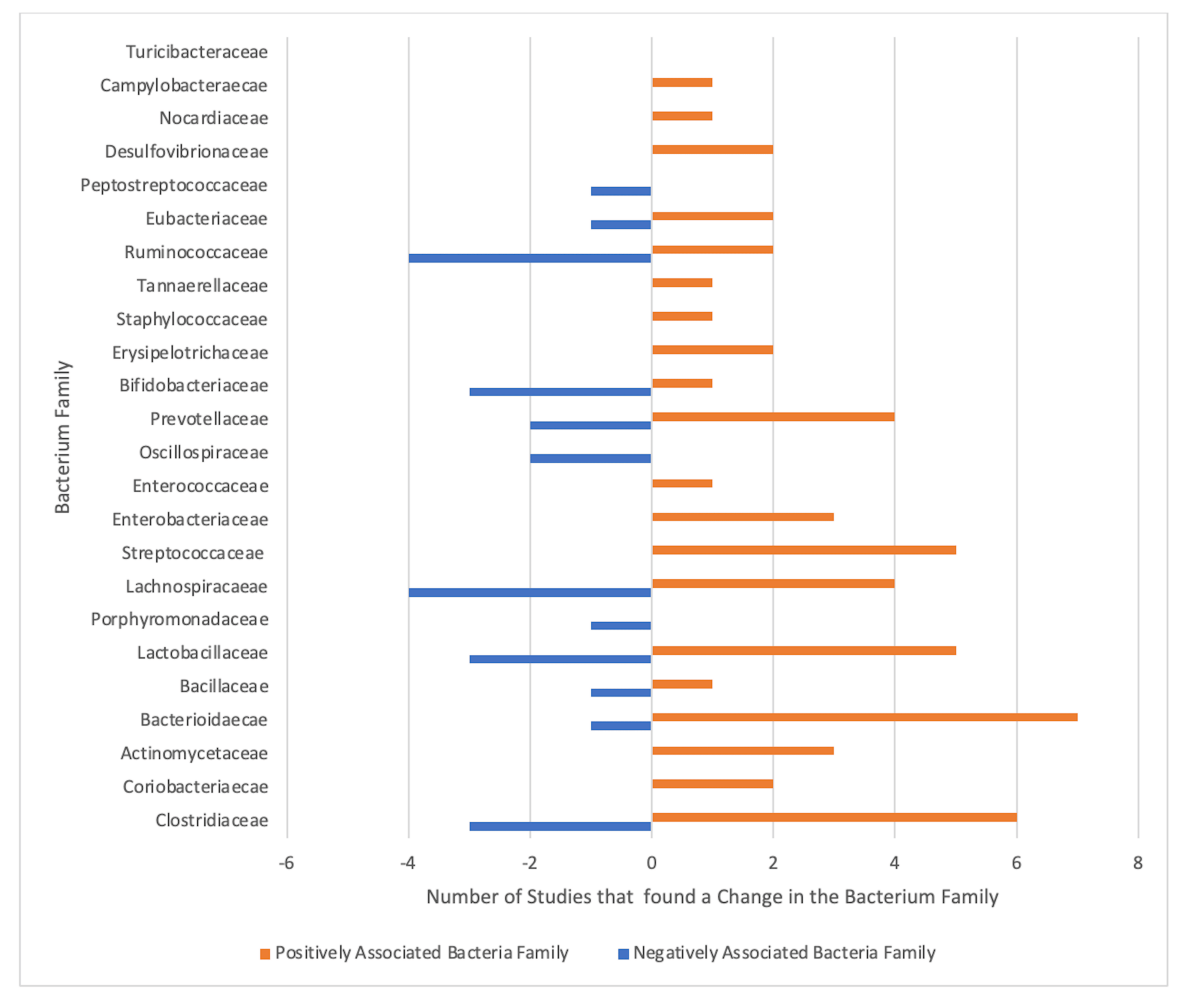
Number of studies displaying positive or negatively associated bacterium families with systemic lupus erythematosus patients. Orange: positively associated bacteria family; blue: negatively associated bacteria family. The negative number of studies represents the absolute value of studies found with negatively associated bacterium families.

Positively identified bacteria can be defined as the microbiota in the gut that was significantly increased in SLE patients (or animals) when compared to control groups without SLE. Negative bacteria can be defined as the microbiota in the gut that was significantly decreased in SLE patients and animals when compared to the control groups. The comparison of these studies is limited due to the differences in reporting of the gut microbiota within SLE patients; some studies reported differences in the bacterial phylum, while others reported bacteria families, genera, or species. However, with consideration of these limitations, the consistency of the findings prompts our discussion of the changes in the gut microbiota and the dysbiosis effects on the symptomatology of SLE. The number of articles that found each microbiota family to be elevated or depressed is depicted in Figure 2.

Discussion

Positively Associated Bacteria With SLE

*Lactobacillaceae*: The literature suggests various microorganisms play a role in the development of SLE via gut microbiomes. One of several microorganisms proposed to have a positive correlation with the development of SLE is *Lactobacillus*. Xiang et al. suggested that *Lactobacillus* plays a role in multiple autoimmune diseases, including SLE, type 1 diabetes, and Grave’s disease [8]. Researchers have posited various factors, including the ability to propagate inflammation, as one of the main mechanisms of pathogenesis. In addition, studies suggested that patients with SLE had diminished diversity in feces compared to patients who were healthy controls [17]. Liu et al. found that SLE-positive patients exhibit reduced diversity, with a majority of *Lactobacillus* found in fecal matter [17]. However, contrary to findings in a paper published by Luo et al., the severity of the disease did not correlate with a change in the diversity or abundance of microorganisms found in the feces [14]. Contrastingly, Ma et al. showed no change in diversity between SLE patients and healthy controls; however, they posited that the susceptibility of SLE was due to the abundance and appearance of specific species, including *Lactobacillus* [21]. Furthermore, current literature elucidates an increase in susceptibility to SLE in mice that have gut dysbiosis concentrated with organisms such as *Lactobacillus*, which appear to alter the metabolism of tryptophan. Specifically, *Lactobacillus reuteri* and *L. johnsonii* were found to be abundant in mouse models susceptible to SLE. Similarly, Luo et al. found an increase in *Lactobacillus* species, specifically *L. reuteri* in gut dysbiosis that could be correlated with more severe disease measured by hemolytic anemia, proteinuria, and antinuclear antibodies [14]. Choi et al. and Ma et al. posit that certain microorganisms can alter the metabolism of specific amino acids, which could create greater susceptibility to SLE [11,21]. Choi et al. found an alteration of tryptophan metabolism, while Ma et al. discovered an increase in histidine in the gut microbiome of patients with SLE [7,16]. Overall, these studies suggest that Lactobacillaceae may play a role in the pathogenesis of SLE. 

*Clostridiaceae*: *Clostridium* has been correlated with a positive and negative influence on the susceptibility and progression of SLE. Luo et al. suggest that *Clostridium* is one of several bacteria that were found in abundance in the pre-disease to diseased state of SLE [14]. Specific species of *Clostridium* are more abundant in patients with diagnosed SLE. These include ATCC BAA-442 and *Clostridium leptum*. Chen et al. suggested that *Clostridium* species were among those with the strongest correlation to the Systemic Lupus Erythematosus Disease Activity Index (SLEDAI) score [7]. Furthermore, *C. nexile* was among the top species enriched in the microbiota in patients with lupus nephritis, and the oral microbiota of SLE patients was found to have similarly high levels as found in the gut compared to control [7]. Therefore, regulating the levels of *Clostridium* species within SLE patients may offer therapeutic benefits by decreasing the severity of the disease state. 

*Lachnospiraceae* and *Ruminococcaceae*: *Lachnospiraceae* and *Ruminococcaceae* are both part of the family of butyrate-producing anaerobes [26]. The levels and roles of both bacteria in SLE patients were controversial among the studies included in this review. Regardless, while their levels require a deeper level of investigation, it can be concluded that both bacterial families are significantly affected by the SLE disease process. In mouse studies, the entire *Lachnospiraceae* family seemed to be elevated in SLE. However, in humans, *Blautia* was the predominant genus of *Lachnospiraceae* that was elevated [14]. Specifically, *Ruminococcus gnavus* was found to be significantly elevated in SLE patients. Although named for the *Ruminococcaceae* family, this bacterium has been reassigned to the *Blautia* genus of the Lachnospiraceae family [27]. Toumi et al. found that *Ruminococcaceae*, *Bifidobacterium*, and *R. gnavus* may play an essential role in SLE severity [13]. One proposed mechanism underlying pathogenesis considers the standard role of *Lachnospiraceae* and *Ruminococcaceae* in the gut microbiome. Both bacteria are members of a group of gut microbiota referred to as butyrate-producing bacteria for their ability to make a short-chained fatty acid called butyrate, a vital component of the gut-brain axis [28]. One of the main functions of butyrate outlined by Trompette et al. is its ability to alter the mitochondrial metabolism of epidermal keratinocytes and produce structural components to strengthen the skin barrier function [29]. Concerning SLE, two main mechanisms of disease involving the *Lachnospiraceae* and Ruminococcaceae families might be at play. These mechanisms were proposed in lupus-prone mice. First, it is proposed that when there are deficiencies in Fas signaling in the disease, it impairs butyrate-induced apoptosis of T-cells, which impairs the ability of *Lachnospiraceae* to suppress inflammation in lupus-prone mice and leads to uncontrolled proinflammatory T-cell responses [12]. The dysregulation of butyrate due to abnormal microbiota levels may be correlated with the cutaneous role butyrate plays in the skin barrier and, thereby, sensitivity to sunlight and/or environmental allergens. Thus, these changes may lead to skin manifestations of SLE, such as malar rash, discoid rash, and photosensitivity. This is further supported by the evidence that topical hydrocortisone butyrate is used to treat cutaneous lupus erythematosus, indicating the re-addition of butyrate to the body may be useful in the therapeutic management of dermatomal manifestations [30]. The second mechanism of disease, involving *Lachnospiraceae*, may impair lipid and glucose metabolism, which could contribute to inflammation [31]. As support, elevated levels of *Lachnospiraceae* are seen in early onset type 1 diabetes and may be related to type 2 diabetes as well [31]. Likewise, there may be a pro-inflammatory effect of *Lachnospiraceae* in SLE. Further research should be conducted to compare SLE patients with decreased levels of *Ruminococcaceae* or *Lachnospiraceae* versus elevated levels to determine a stronger correlation between bacterial levels and the disease process. Additional investigation could also explore the differences in the skin manifestations and symptoms experienced.

Negatively Associated Bacteria With SLE

*Bifidobacteriaceae*: Three of the articles included negative associations associated with SLE patients and *Bifidobacterium* in their gut microbiomes. Engevik et al. found that *Bifidobacterium dentium* can adhere to intestinal mucus and secrete metabolites, which can then modulate goblet cell function and upregulate major mucin 2 (MUC2), which can work as a therapeutic agent for intestinal dysfunction [32]. Therefore, a decrease in *Bifidobacterium* might result in goblet cell dysfunction and lowered mucus secretion. Hensel et al. support this idea. In a study of SLE patients, they found a significant decrease in the intensity of MUC2 immunofluorescence staining, which indicates an alteration of the function of goblet cells or the secreted mucus [33]. By treating the dysbiosis of *Bifidobacterium* in SLE patients, there is a potential to restore goblet cell function and mucus secretion and offer therapeutic relief for the intestinal dysfunctions these patients may be experiencing. In a study conducted by Fu et al., there was a strong association between omega-3 fatty acid supplementation and the growth of the *Bifidobacterium* [34]. In addition, it appears that omega-3 supplementation may help lower disease [35]. With omega-3 administration, either by supplementation of fish oil or dietary fish, the *Bifidobacterium* microbiota could be restored in SLE patients. 

*Firmicutes*/*Bacteroidetes* ratio: Many studies concluded that in both animal and human studies, there is a lower *Firmicutes*/*Bacteroidetes* ratio compared to control. This ratio has been significant in several gut dysbiosis diseases over the past decade, including obesity. In obese patients, this ratio is typically over-elevated compared to normal-weight patients [36]. The mechanism behind this ratio is thought to involve *Firmicutes*, which are much more effective at absorbing energy by digesting polysaccharides than *Bacteroidetes*, which is why they appear to dominate in the microbiome of obese patients [36]. Additionally, the microbiota is involved in the storage of triglycerides in adipose tissue by inhibiting the release of fasting-induced adipose factor (Fiaf), which inhibits lipoprotein lipase [36]. Thereby, with the significant increase in this ratio in the obese population, it is proposed that there is an increase in the storage of fats in adipose tissue. However, when this ratio is significantly decreased, there is the potential for the reverse mechanism, with a decrease in fat storage and carbohydrate metabolism. SLE is a disease that varies significantly in symptomatology amongst patients, but the most common symptoms include fever, malaise, arthralgias, myalgias, headache, and loss of appetite and weight [37]. The lowered *Firmicutes*/*Bacteroidetes* ratio could explain why many SLE patients have a loss of appetite and weight as well as fatigue and headaches. By raising this ratio through dietary means and/or probiotics, there may be potential to relieve the gastrointestinal symptoms SLE patients experience. There are limitations to this theory as these symptoms are most likely multifactorial, and a high-fat diet and obesity have been reported to potentially play a role in the pathogenesis of autoimmune diseases such as SLE.

## Conclusions

This review set out to summarize the primary research on the changes in the gut microbiome in SLE patients. The results showed that the gut microbiome was significantly disturbed, with a much lower diversity, in SLE patients compared to the control. Within each study, some bacteria demonstrated a significantly increased bacterial load, while other bacteria were found in significantly decreased numbers. Furthermore, some of the studies showed a causal relationship between the changes in the gut microbiota and the onset of SLE symptoms. Based on the established role of these bacteria within the gut microbiome, the disruption in the gut ecosystem could explain the symptomatology common in SLE patients. By addressing these changes, our scoping review encourages further research in establishing a true causal relationship between the bacterial changes in SLE patients as well as furthering the scope of microbiota changes in other systems as well as other autoimmune diseases.
