# Correction: A Scoping Review of the Positive and Negative Bacteria Associated With the Gut Microbiomes of Systemic Lupus Erythematosus Patients

**DOI:** 10.7759/cureus.c255

**Published:** 2025-08-24

**Authors:** Marissa N McPhail, Michael Wu, Kelsey Tague, Hassaan Wajeeh, Michelle Demory Beckler, Marc M Kesselman

**Affiliations:** 1 Osteopathic Medicine, Nova Southeastern University Dr. Kiran C. Patel College of Osteopathic Medicine, Fort Lauderdale, USA; 2 Microbiology and Immunology, Nova Southeastern University Dr. Kiran C. Patel College of Allopathic Medicine, Fort Lauderdale, USA; 3 Rheumatology, Nova Southeastern University Dr. Kiran C. Patel College of Osteopathic Medicine, Davie, USA

This article has been corrected to fix the following typo in the PRISMA diagram: "Duplicate records removed (n = **26**)" has been corrected to "Duplicate records removed (n = **23**)".

